# Messenger RNA Turnover Processes in *Escherichia coli, Bacillus subtilis*, and Emerging Studies in *Staphylococcus aureus*

**DOI:** 10.1155/2009/525491

**Published:** 2009-03-05

**Authors:** Kelsi L. Anderson, Paul M. Dunman

**Affiliations:** Department of Pathology and Microbiology, University of Nebraska Medical Center, Omaha, NE 68198-6495, USA

## Abstract

The regulation of mRNA turnover is a recently appreciated phenomenon by which bacteria modulate gene expression. This review outlines the mechanisms by which three major classes of bacterial *trans*-acting factors, ribonucleases (RNases), RNA binding proteins, and small noncoding RNAs (sRNA), regulate the transcript stability and protein production of target genes. Because the mechanisms of RNA decay and maturation are best characterized in *Escherichia coli*, the majority of this review will focus on how these factors modulate mRNA stability in this organism. However, we also address the effects of RNases, RNA binding proteins, sRNAs on mRNA turnover, and gene expression in *Bacillus subtilis*, which has served as a model for studying RNA processing in gram-positive organisms. We conclude by discussing emerging studies on the role modulating mRNA stability has on gene expression in the important human pathogen *Staphylococcus aureus*.

## 1. Introduction

RNA steady-state levels are a function of both transcript synthesis and decay. Nonetheless, studies of prokaryotic mRNA regulation have historically interpreted changes in mRNA titers on the basis of transcript synthesis alone. It has been recently recognized that this is likely an oversimplification; the modulation of mRNA decay also profoundly affects mRNA titers and, consequently, protein production. A growing body of literature suggests that the regulation of mRNA turnover may be a ubiquitous phenomenon spanning many, if not all, bacterial species. 

As a precursor to reviewing the factors that regulate bacterial mRNA turnover, we provide examples of how alterations in transcript degradation mediate bacterial adaptation to stress conditions, growth phase transition, and pathogenesis. This is designed to provide the reader with an appreciation of the magnitude of biological responses that are regulated at the level of mRNA turnover and introduce three classes of molecules that govern these changes: ribonucleases (RNases), RNA binding proteins, and noncoding RNAs (sRNA). 

Bacteria have developed highly orchestrated responses to environmental stress which, when elicited, alter the cellular physiology in a manner that enhances survival. Recent reports indicate that regulated changes in mRNA turnover play a vital role in bacterial stress adaptation (reviewed in [1]). For instance, the *Escherichia coli* cold shock protein CspA, which resolves low temperature mediated mRNA secondary structures that would otherwise impede translation, reaches more than 10% of the total cellular protein concentration during cold shock conditions [2–4]. Upon temperature downshift, increased *cspA* mRNA stability, as opposed to changes in transcript synthesis, primarily accounts for the increase in CspA production [5–7]. This change in *cspA* mRNA stability reflects alterations in the transcript's vulnerability to digestion by the endoribonuclease RNase E [8]. As described below, regulating a target transcript's accessibility to ribonucleases is a common means of modulating mRNA turnover. Stress-responsive changes in mRNA turnover are not restricted to *E. coli*. The *Vibrio angustum* response to nutrient deprivation and the *Klebsiella pneumoniae* nitrogen fixation system are mediated by alterations in mRNA degradation [1, 9, 10]. Similarly, stringent, cold shock, and heat shock conditions alter *Staphylococcus aureus* mRNA turnover [11]. 

In addition to stress adaptation, the modulation of mRNA turnover may play a role in regulating bacterial cell growth phase processes. Indeed, the transition from log to stationary phase growth stabilizes many transcripts within the human pathogens *S. aureus* and *Streptococcus pyogenes* (Anderson & Dunman, unpublished; [12]). While it remains to be seen what, if any, biological significance growth phase-induced alterations in mRNA turnover might have on these organisms, the effects of growth dependent changes in transcript stability are well characterized for other bacterial species. For instance, stability of the *E. coli* outer membrane protein A (*ompA*) transcript is inversely correlated with growth rate [13–15]. As described further below, ribonuclease E, the RNA binding protein Hfq, and a noncoding RNA molecule MicA/SraD coordinately regulate *ompA* mRNA stability which, in turn, influences OmpA production [13–15]. 

The modulation of mRNA turnover also mediates bacterial virulence factor production. Although several examples exist, perhaps the best includes the effector of the *E. coli* carbon storage regulatory system, CsrA. CsrA binds target transcripts and alters the mRNA stability and, consequently, translation of proteins involved in carbon utilization, biofilm formation, and motility [16–18]. Interestingly, the effects of CsrA binding are transcript specific. For instance, binding stabilizes the *flhDC* transcript and leads to increased production of proteins involved in flagellum biosynthesis. This increase in protein production may result from either activation of translation or protection of the message from ribonuclease attack [17]. Conversely, CsrA binding to the *pgaABCD* leader sequence results in inhibition of ribosome binding, transcript destabilization, and, consequently, loss of polysaccharide adhesion (PGA) production which is required for biofilm formation and plays a role in pathogenesis [16]. CsrA homologues influence virulence factor production in *Salmonella typhimurium*, *Erwinia carotovara* ssp. *carotovora*, and *Pseudomonas aeruginosa* [19–21]. Recent reports have also linked *S. aureus'* regulation of virulence factor mRNA turnover to corresponding changes in virulence factor protein production. For instance, a product of the pleiotropic regulatory locus, *sarA*, appears to stabilize the mRNA and repress production of two *S. aureus* virulence factors: protein A (*spa*) and collagen adhesion protein (*cna*) [22]. Likewise, Romby et al. have shown that a sRNA-like molecule, RNAIII, and endoribonuclease III modulate the mRNA turnover of a subset of the organism's virulence factors [23, 24]. Although less characterized, production of other *S. aureus* virulence factors, fibronectin binding protein B (*fnbB*) and coagulase (*coa*), is also mediated by mRNA turnover [25].

Admittedly, studies designed to assess the effects of mRNA turnover on protein production are in their infancy. Nonetheless, the aforementioned examples establish that regulated transcript degradation, in part, modulates many biological processes. Although the molecular components that govern the stabilization/destabilization of individual transcripts differ, pioneering work from the Deutscher, Condon, Romeo, and Gottesman laboratories indicates that they can be broadly categorized as ribonucleases (and auxiliary factors), RNA binding proteins, and small noncoding RNAs. In this review, we will overview these three major classes of *trans-*acting RNA turnover regulatory molecules in the prototypic gram-negative and gram-positive bacterial species, *E. coli* and *Bacillus subtilis,* respectively. Finally, we describe recent *S. aureus* RNA turnover studies and provide an emerging view of how these factors may contribute to the organism's ability to coordinately regulate virulence factors and cause disease.

## 2. Control of *Escherichia coli* Messenger RNA Degradation

### 2.1. Ribonucleases

Ribonucleases (RNases) are a class of enzymes that are responsible for RNA degradation and processing. These enzymes are classified as endo- and 3′ → 5′ exoribonucleases (Table 1). In addition, an RNase with unique 5′ → 3′ exoribonucleolytic activity has recently been described in *B. subtilis* [26, 27]. At least two ribonucleases are components of a holoenzyme complex, the RNA degradosome, which catalyzes bulk *E. coli* mRNA degradation. In addition to the RNA degradosome, *E. coli* produces other endo- and exoribonucleases, many of which are largely considered to be involved in rRNA and tRNA maturation rather than bulk mRNA decay. A subset of these enzymes contribute to the decay of individual mRNA species, whereas others have not yet, or have only circumstantially, been linked to mRNA degradation. It is likely that as each of these ribonucleases becomes better characterized, many will be found to contribute to mRNA decay. In the pages that follow, we describe components of the *E. coli* RNA degradosome and other RNases that mediate processing/degradation of targeted bacterial mRNA species. Moreover, we discuss several mechanisms by which the activity of the degradosome and other bacterial RNases is modulated as a means of regulating mRNA levels during cellular proliferation and stress conditions. 

#### 2.1.1. The Degradosome

**Figure 1:**
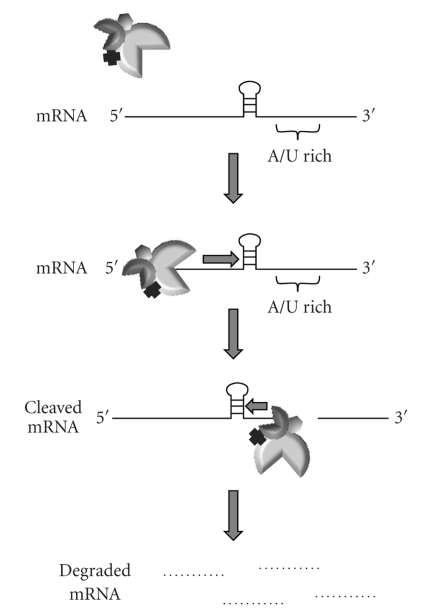
Degradosome-mediated RNA decay. The *E. coli* degradosome is composed of at least four subunits: RNase E, PNPase, RhlB helicase, and enolase. The initial RNA cleavage event is catalyzed by the 5′ → 3′ endoribonuclease RNase E (large cut-out circle) which loads onto a transcript and scans for downstream cleavage sites: A/U rich regions proceeded by stem-loop structures in 5′ monophosphorylated transcripts. The 3′ → 5′ exoribonuclease PNPase (small cut-out circle) catalyzes cleavage of RNase E-generated decay intermediates. Otherwise inhibitory secondary structures to PNPase-mediated degradation are resolved by RhlB helicase (cross). The role of enolase (hexagon) in mRNA decay is not well characterized.

The *E. coli* degradosome is composed of at least four proteins: ribonuclease E (RNase E), polynucleotide phosphorylase (PNPase), RhlB RNA helicase, and the glycolytic enzyme enolase [28]. As shown in Figure 1, a model for mRNA decay has been proposed in which the degradosome loads and scans an mRNA molecule for an RNase E cleavage site, A/U-rich sequences usually proceeded by a stem-loop structure in 5′ monophosphorylated transcripts, in the 5′ → 3′ direction [29, 30]. Once this site is encountered, RNase E catalyzes an initial endoribonucleolytic event and then continues to cleave the transcript at additional downstream target sites. Fragmentation products are subsequently degraded by the 3′ → 5′ exoribonuclease, PNPase [31]. RhlB RNA helicase-mediated removal of mRNA secondary structures is thought to facilitate PNPase degradation [32], whereas enolase may participate in bulk degradation of metabolic enzyme transcripts [28, 33].

Components of the degradosome are localized to the cell membrane and are organized into helical filaments that coil around the length of the cell [34–36]. This organization may provide a means for the apparatus to interact with other cell membrane-associated macromolecular complexes [35, 36]. Indeed, the degradosome appears to be a dynamic organelle; during cold shock conditions, the complex's RNA helicase is replaced by an alternative cold shock RNA helicase, CsdA [37]. The cold shock protein CspE also interacts with degradosome-associated ribonucleases [38]. Further, the heat shock proteins GroEL and DnaK have been shown to be associated with the degradosome [39]. It remains to be seen whether these auxiliary factors affect global mRNA decay. Rather, it seems likely that they may redirect the efficiency with which the degradosome catalyzes turnover of individual or subsets of mRNA species. This would provide an efficient means of modulating protein production in a manner that allows cells to quickly adapt to otherwise deleterious conditions. It is very likely that as the field matures, additional degradosome auxiliary factors will be identified and characterized. As a first step toward understanding how the holoenzyme's function can be altered in response to endogenous and exogenous cues, one must first appreciate the components of the “native” RNA degradosome complex. 

RNase E [*rne*; 118 kilodalton (kDa)] is an essential endoribonuclease that organizes other components of the *E. coli* degradosome and initiates bulk RNA decay [31]. The C-terminal region of RNase E acts, in part, as a scaffold for assembly of the other major degradosome components [39–41]. The protein's internal domain is required for cell membrane association, whereas its N-terminus is required for cell viability and RNA cleavage [31, 41, 42]. In addition to its role in mediating bulk mRNA degradation, RNase E is involved in the maturation of both ribosomal and transfer RNA molecules [43–46]. 

Because RNase E is responsible for many RNA decay and maturation processes, it stands to reason that it must be tightly regulated. Indeed, RNase E autoregulates itself by controlling the cleavage of its cognate mRNA [47, 48]. When RNase E activity is low or when substrate transcripts reach high levels, the rate of *rne* cleavage is reduced which results in increased RNase E production [49]. As substrate molecules are depleted, *rne* mRNA degradation and protein production return to basal levels [49]. As discussed further below, *trans*-acting factors such as noncoding RNAs, RNA binding proteins, and the translation apparatus frequently indirectly affect RNase E function by altering a target transcript's accessibility to the enzyme.

During normal laboratory growth conditions, polynucleotide phosphorylase (PNPase; *pnp*; 80 kDa) functions as a nonessential, 3′ → 5′ exoribonuclease component of the degradosome [50]. Although other cellular exoribonucleases can rescue a loss of PNPase activity, they do so with reduced efficiency; *pnp*-mutants produce transcripts with mildly increased steady-state levels [51]. As opposed to normal growth conditions, PNPase is essential for survival at low temperatures (<20°C). Following cold acclimation, the enzyme is required for degradation of low temperature stabilized transcripts whose accumulation would otherwise be lethal [52]. The temperature mediated change in cellular PNPase dependence suggests that ribonuclease functions/importance changes in response to internal and/or external stimuli. As the field matures, it is likely that this phenomenon will be observed for additional RNases. In addition to its role in degradosome-mediated RNA degradation and cold shock adaptation, PNPase may also participate in 3′ polyadenylation of mRNA [50, 53, 54]. 

Although not ribonucleases, RhlB helicase and enolase are integral members of the *E. coli* degradosome. RhlB (*rhlB*; 47 kDa) is a DEAD box RNA helicase that unwinds RNA secondary structures via energy generated by ATP hydrolysis [55]. Presumably, RhlB facilitates PNPase-mediated digestion of RNase E-generated fragments [32]. The glycolytic enzyme enolase (*eno*; 46 kDa) is an abundant *E. coli* protein; ~10% of all cellular enolase is associated with the degradosome [33]. Although the function of enolase as part of the degradosome has not been elucidated, some studies have indicated a possible role for enolase in bulk mRNA turnover of some metabolic enzymes [28, 33]. 

#### 2.1.2. Endoribonucleases

RNase III (*rnc*; 26 kDa) is an endoribonuclease that cleaves double-stranded RNA [56]. Although the enzyme is best known for its role in rRNA maturation, RNase III also regulates the mRNA decay of a subset of RNA species, including *pnp*, which contain a 5′ stem loop structure. Other noted RNase III substrates include the intergenic regions of *rplL*-*rpoB* [57], *rpsO*-*pnp* [58, 59], *dicA*-*dicF*-*dicB* [60], and *metY*-*nusA* [61] transcripts. In addition to its role in degrading target RNA molecules, RNase III has been shown to bind the 5′ untranslated region (UTR) of bacteriophage *λ* cIII transcripts. Binding alters the mRNA conformation and alleviates an otherwise translation-inhibitory structure [62, 63]. Thus, RNase III has at least two post-transcriptional regulatory mechanisms which are facilitated by its RNA binding and/or RNA degradation activities.

RNase P is a holoenzyme consisting of a ribozyme (*rnpB*; 377 nucleotides) and at least one protein subunit, RnpA (*rnpA*; 14 kDa) [64]. The major function of the ribonucleoprotein complex has been considered to be catalyzing cleavage of the 5′ leader sequence of precursor tRNAs [65, 66]. It is well established that the ribozyme is the catalytic unit, whereas the protein component aids in substrate recognition [65, 67]. Interestingly, although both the RNase P ribozyme and protein subunits are essential in vivo, the protein subunit is not required for in vitro precursor tRNA processing [65]. RNase P has also been implicated in the cleavage of intergenic regions of polycistronic mRNA molecules and the degradation of guide RNAs [68, 69]. Thus, RNase P may facilitate both tRNA maturation and degradation of subsets of mRNA species during distinct conditions. Indeed, RNase P activity is regulated in response to nutrient limitation [70, 71].

Two ribonuclease H genes exist within *E. coli*. Although they have similar functions, they share limited sequence similarity. RNase HI (*rnhA*; 18 kDa) was the first to be identified. The enzyme degrades the RNA component of RNA/DNA duplexes [72]. A precise physiological role has not been determined for RNase HI. However, potential functions have been proposed including the removal Okazaki fragment primers as well as primers at sites other than the vegetative origin of replication [72, 73]. The second *E. coli* ribonuclease H gene, RNase HII (*rnhB*; 23 kDa), also degrades the RNA component of RNA/DNA hybrid molecules [74, 75]. Like RNase HI, the enzyme's biological function is unknown. 

RNase G (*rng/cafA*; 55 kDa) was initially termed CafA because it was first determined to be involved in cell division and the formation of cytoplasmic axial filaments [46]. CafA shares N-terminal amino acid homology with RNase E, thus, it was not surprising when the protein was found to exhibit endoribonuclease activity with specificity to A/U-rich sequences and was subsequently renamed RNase G. Despite the similarities to RNase E, RNase G is not essential and is not responsible for bulk *E. coli* mRNA decay [76]. Nonetheless, RNase G appears to affect the mRNA turnover of at least two transcripts: fermentative aldehyde dehydrogenase (*adhE*) and enolase (*eno*) [76–78]. 

RNase BN/Z (*elaC*; 33 kDa) is a nonessential endoribonuclease in *E. coli*. In other organisms, the enzyme cleaves CCA-less tRNA molecules endonucleolytically [79, 80]. However, all *E. coli* tRNAs have chromosomally encoded CCAs and thus are not cleaved by RNase Z. Nonetheless, RNase Z is able to mature tRNAs in the absence of the other 4 tRNA maturation exoribonucleases (see below) [81]. Furthermore, the steady-state levels of over 150 transcripts increased in the absence of RNase Z including *rpsT, cspE, htpG, glpQ,* and *adhE* [82].

RNase LS (*rnlA*; 40 kDa) was initially identified as a regulator of bacteriophage T4 late gene silencing, which is responsible for degrading the corresponding transcripts [83]. RNase LS also moderately affects the turnover of several *E. coli* transcripts and profoundly affects the stability of *bla* and an accumulated fragment of 23S rRNA [83]. It has been suggested that RNase LS exists in a multiprotein 1000 kDa complex which indicates that the activity of the enzyme is dependent on interactions with other proteins [84]. 

RNase I (*rna*; 29 kDa) is a nonessential and nonspecific endoribonuclease that resides in the *E. coli* periplasmic space which provides a unique mechanism by which the enzyme's activity can be tightly regulated. During nonstress conditions, the cytoplasmic concentration of RNase I is presumably low. However, during stress-induced transcriptional arrest, RNase I leaks from the periplasmic space into the cytoplasm where it rapidly degrades rRNA and tRNA [85]. Variants of RNase I (RNase M and RNase I*) are present in *E. coli* and have been shown to degrade mRNA [86, 87]. 

Expression of chromosomally encoded toxin-antitoxin systems enables cells to rapidly shut down cellular processes in response to changes in growth conditions [88, 89]. These bicomponent systems consist of a short-lived antitoxin and a stable toxin. Under normal growth conditions, the antitoxin silences the toxin. However, in stress-inducing environments when cellular processes are downregulated, the antitoxin is rapidly degraded resulting in derepression of the toxin. Studies on the targets of the toxin components of these systems have indicated some function as ribonucleases (Table 1; [88, 89]). There are two classes of toxin-mediated ribonucleases in *E. coli*: (1) toxins that cleave mRNA molecules present in ribosomes which include RelE [90] and YoeB [91] and (2) toxins that cleave mRNAs independent of translation which includes MazF [92–94] and ChpBK [95]. Both classes of toxin-mediated ribonucleases inhibit translation and, consequently, protein production by degrading target transcripts.

#### 2.1.3. Exoribonucleases

Seven *E. coli* exoribonucleases have been identified (Table 2). Of these PNPase, RNase II, RNase R, and Oligo-RNase are established to affect mRNA degradation and, consequently, protein production. The remaining exoribonucleases RNase PH [96, 97], RNase D [98], and RNase T [99]. are believed to function primarily as tRNA maturation enzymes; none have been identified to modulate mRNA turnover. However, it is important to recognize that formal studies designed to globally measure what effect, if any, these enzymes have on mRNA turnover have not been described. Thus, one cannot rule out the possibility that they may also affect mRNA degradation, and until proven otherwise, it is possible that virtually any defined ribonuclease may play a role in the mRNA turnover of individual or subsets of bacterial transcripts. 

RNase R (*rnr*; 95 kDa) is a processive 3′ → 5′ exoribonuclease that cleaves structured polyadenylated [poly(A)] mRNA, tRNA, and rRNAs in vitro [100, 101]. Thus, it is thought that the in vivo role of RNase R is to degrade highly structured RNA molecules, such as those containing repetitive extragenic palindromic sequences which are associated with stable stem-loops [101]. RNase R can degrade these secondary structures in the absence of an RNA helicase provided there is a 3′ single-stranded region, such as a poly(A) tail, available for the enzyme to bind and initiate decay [101]. RNase R activity increases in response to several stress conditions including entry into stationary phase, starvation, and cold shock [102, 103]. During these conditions, RNase R has been proposed to catalyze degradation of structured RNA molecules when protein production needs to be stalled [102]. 

RNase II (*rnb*; 72 kDa) is a processive 3′ → 5′ exoribonuclease that accounts for ~90% of all exoribonucleolytic activity of poly(A) RNA [51, 104]. The enzyme processes the 3′ tail of immature tRNA [105]. It also regulates the stability of mRNA by removing poly(A) tails which makes them less accessible to the degradosome [51, 106]. 

Oligo-RNase (*orn*; 38 kDa) is an essential, processive 3′ → 5′ exoribonuclease which, as the name implies, cleaves short oligoribonucleotides. The enzyme copurifies with PNPase suggesting that it may catalyze digestion of oligoribonucleotide intermediates generated during PNPase-mediated mRNA decay [107, 108].

### 2.2. RNA Binding Proteins

**Figure 2:**
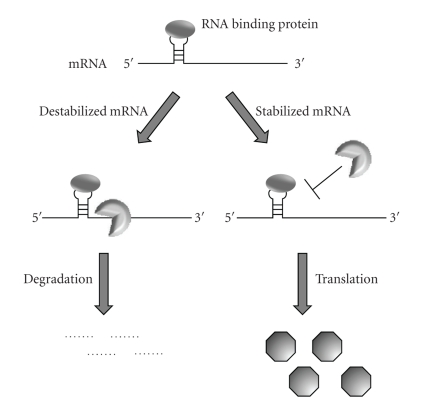
RNA binding proteins affect mRNA stability. RNA binding proteins affect gene expression by stabilizing or destabilizing mRNA targets by altering their susceptibility to RNases. RNA binding proteins may inhibit protein production by destabilizing mRNA molecules which results in RNase-mediated degradation. Alternatively, RNases may be inhibited by RNA binding proteins which stabilizes the mRNA resulting in increased protein production.

Another major class of mRNA turnover regulatory molecules includes RNA binding proteins. As shown in Figure 2, their binding frequently stabilizes or destabilizes mRNA species by affecting the transcripts susceptibility to ribonuclease digestion. Examples of two well-characterized RNA binding proteins are discussed below.

The Host factor I protein (Hfq; 11 kDa) is an RNA binding protein that affects mRNA stability by facilitating base pairing between sRNAs (described below) and their mRNA targets. This, in turn, can increase or decrease a transcript's accessibility to ribonucleases [109–112]. For example, in rapidly growing cells, outer membrane protein A (*ompA*) mRNA is stabilized by elements in its 5′ UTR [113, 114]. However, upon entry into stationary phase, the noncoding sRNA, MicA/SraD, is induced and binds *ompA* mRNA [13, 14]. Hfq binds both *ompA* and the sRNA in vitro and presumably facilitates base pairing between these RNAs in vivo [13, 14]. Pairing inhibits ribosome binding and promotes RNase E-dependent degradation [13, 14]. Additional examples of Hfq-mediated sRNA:mRNA pairing are discussed below (see Section 2.3). In addition to catalyzing RNA degradation, Hfq has been shown to stabilize DsrA, RyhB, and OxyS transcripts, all of which are well-studied sRNAs. In these cases, Hfq binding overlaps with RNase E cleavage sites thereby reducing the transcripts' accessibility to ribonuclease attack [115]. In addition to its role in facilitating sRNA:mRNA pairing, Hfq stabilizes transcripts by enhancing the PAP I-mediated elongation of poly(A) tails in vivo and in vitro [116]. 

The carbon storage regulator protein (CsrA; 7 kDa) is a negative regulator of postexponentially induced metabolic pathways; a positive regulator of glycolysis, acetate metabolism, and motility; and a repressor of biofilm formation in *E. coli* [16–18]. The protein's regulatory effects are, in part, due to its ability to modulate the mRNA turnover of target transcripts. This has been best characterized for the polycistronic glycogen biosynthesis transcript, *glgCAP*. During exponential phase growth when nutrient sources are readily available, CsrA binds to the 5′ UTR of *glgCAP* mRNA which, in turn, inhibits ribosome loading and promotes transcript degradation [18, 117, 118]. During stationary phase growth, glycogen biosynthesis is upregulated, in part, because CsrA no longer efficiently binds *glgCAP* transcripts. Rather, the protein becomes predominantly sequestered into a ribonucleoprotein complex comprised of 18 CsrA subunits and one small RNA, CsrB [119].

Although not as extensively characterized, CsrA also seems to regulate the transcript stability and, consequently, protein production of several virulence factors [16, 17]. However, the effects of CsrA binding appear to be transcript specific. For instance, CsrA decreases the half-life of *pgaABCD* which prevents PGA (poly-beta-1,6-N-acetyl-d-glucosamine) production; a cell surface polysaccharide that promotes biofilm formation [16]. Thus, CsrA's mechanism of action may be similar to its role in *glgCAP* regulation; binding may inhibit translation initiation and increase ribonuclease degradation [16]. Conversely, CsrA stabilizes the flagellar transcriptional activator genes *flhDC*, presumably by binding to the transcript [17]. Thus, CsrA promotes motility either by acting as an activator of translation or by protecting the transcript from ribonuclease digestion [17]. 

As described above, Hfq and CsrA are two well-characterized *E. coli* RNA binding proteins that influence protein production by altering mRNA stability. In addition, the histone-like protein H-NS regulates mRNA stability by binding to target transcripts [120]. Other H-NS like proteins regulate mRNA stability as well [121]. Further studies will likely identify additional RNA binding proteins that regulate gene expression by altering mRNA stability.

### 2.3. Small RNAs

**Figure 3:**
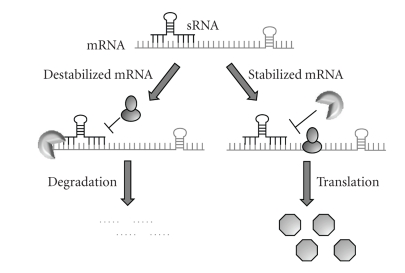
Small RNAs base pair with mRNA targets to affect mRNA stability. Antisense base pairing between sRNAs and their target transcripts mediates mRNA stability by altering the susceptibility of the message to RNases and the translation machinery. Pairing may destabilize mRNA by facilitating RNase-mediated degradation resulting in translation inhibition. In contrast, pairing may stabilize mRNA by inhibiting RNase-mediated degradation resulting in translation of the message and increased protein production.

The third class of regulatory molecules discussed here is small noncoding RNAs (sRNAs). More than 80 sRNAs have been identified in *E. coli*; many of these are components of stress responsive regulons [122, 123]. sRNAs typically do not have a discernable open reading frame encoded in their sequence, thus the RNA molecule rather than a protein product is thought to affect gene expression. As described by the Aiba laboratory, the regulatory effects of sRNAs are mediated largely by their binding to mRNA and affecting translation which, in turn, mediates turnover of target transcripts, as shown in Figure 3 [124]. Other sRNAs such as the ribozyme *rnpB*, tmRNA, and 4.5S regulate gene expression through entirely different processes. *rnpB* processes tRNA molecules and thus affects translation [66], 4.5S is part of the signal recognition particle ribonucleoprotein complex that targets membrane and secreted proteins for translocation during translation [125], and tmRNA is a quality control regulator that rescues stalled ribosomes and facilitates the elimination of proteins whose translation has been prematurely terminated [126]. In the following section, we will overview sRNAs that function as antisense regulatory molecules to influence transcript stability. For more detailed information regarding the identification and other mechanisms of sRNA regulation, we refer the reader to several excellent reviews [122, 123, 127, 128]. 

One of the best-studied sRNAs is DsrA which affects the mRNA turnover of at least two target transcripts: *hns* and *rpoS* [129]. As in the case of the RNA binding protein CsrA, DsrA catalyzes digestion of certain transcripts but stabilizes others. For example, under normal growth conditions, the stationary phase RNA polymerase sigma factor, *rpoS*, transcript is destabilized by the formation of a stable hairpin in its 5′ UTR. Doing so sequesters the ribosome binding site and promotes RNase III-mediated transcript degradation [130]. However, during nonoptimal growth conditions, Hfq catalyzes antisense base pairing between DsrA and *rpoS* enabling efficient translation which stabilizes the message and results in increased protein production [131, 132]. In contrast, DsrA base pairing with the histone-like protein transcript *hns* inhibits ribosome entry which destabilizes the message resulting in decreased H-NS abundance [129].

The ferric uptake regulator, Fur, has classically been considered a repressor protein but also indirectly activates gene expression in response to iron availability via an sRNA. When Fe^2+^ is abundant, Fur becomes activated and inhibits expression of genes involved in various iron acquisition systems [133]. Other genes including those involved in iron storage and intracellular usage are activated by Fur during these same conditions. Massé and Gottesman have shown that Fur-mediated gene activation is indirect and involves the sRNA RyhB [134]. In that study, it was found that Fur represses RyhB synthesis when iron is abundant which, in turn, induces the expression of proteins that bind intracellular iron. However, when iron availability is limited, Fur becomes inactivated resulting in RyhB upregulation and repression of target genes. RyhB represses gene expression by binding to target transcripts in an Hfq-dependent manner which facilitates RNase E-mediated mRNA degradation [135]. Other examples of stress-induced sRNAs include OxyS (oxidative stress; [136]), OmrA/B (osmotic shock; [137]), RprA (cell surface stress; [138]), MicA/SraD (stationary phase; [13, 14]), MicF (oxidative/antibiotic stress; [139]), SgrS (glucose phosphate accumulation; [140]), and Spot 42 (glucose limitation; [141]). 

We do not intend to give the impression that the three classes of molecules discussed above, RNases, RNA binding proteins, and sRNAs, are the sole mediators of bacterial mRNA turnover. In fact, Deborah Steege published an excellent review highlighting the identification, characterization, and cellular role of polyadenylation in bacteria [142]. Poly(A) polymerase I (*pcnB*; 53 kDa) is responsible for adding 10–40 nt poly(A) tails to bacterial RNA species [142, 143]. Although polyadenylated transcripts account for only 0.01–2% of the total cellular mRNA content, polyadenylation plays a significant role in regulating the stability of target transcripts [142, 143]. Indeed, *E. coli* polyadenylated mRNA molecules are rapidly degraded by the 3′ → 5′ exoribonucleases RNase II and PNPase. It is believed that poly(A) tails provide a single-stranded extension region upon which these RNases can bind and initiate decay when otherwise inhibitory secondary structures are present [142, 144]. For example, increased polyadenylation due to overexpression of *pcnB* in *E. coli* destabilizes *rpoS, trxA, lpp, ompA,* and total RNA [143]. Although polyadenylation promotes bacterial mRNA decay, the presence of these elements may also recruit poly(A)-binding proteins, such as CspE, which when bound to poly(A) tails interfere with RNase activity [38]. Nonetheless, RNase E can remove poly(A) tails by endoribonucleolytic cleavage which may eliminate inhibitory poly(A) binding proteins [145]. As mentioned above, RNase II degrades polyadenylated mRNAs; however, polyadenylated *rpoS* is stabilized by RNase II [106]. In vivo, RNase II shortens the *rpoS* poly(A) tail making the transcript less susceptible to PNPase-mediated decay [106].

Collectively, ribonucleases, RNA binding proteins, and noncoding RNA molecules dynamically regulate *E. coli* gene expression by affecting mRNA stability. As will become evident (discussed below), these factors likely modulate mRNA stability in the model gram-positive organism *B. subtilis* and the human pathogen *S. aureus* as well. 

## 3. Control of *Bacillus subtilis* Messenger RNA Degradation

While studies of the mechanism(s) of *E. coli* mRNA degradation are still in their infancy, even less is known about the factors that affect gram-positive bacterial mRNA turnover, even within the model organism *B. subtilis.* Here, we will overview the similarities and differences between *B. subtilis* and *E. coli* mRNA turnover factors.

*E. coli* and *B. subtilis* share several ribonuclease sequence homologues, whereas others are unique to each organism (Table 1). Particularly striking is the absence of a *B. subtilis* sequence homolog to the major component of the *E. coli* RNA degradosome RNase E, which, in turn, has delayed characterization of a *B. subtilis* RNA degradosome. The lack of an RNase E homolog is not specific to *B. subtilis*, rather it is a common characteristic among gram-positive organisms with low G-C content. Nonetheless, two ribonucleases, RNase J1 (*rnjA;* 61 kDa) and J2 (*rnjB*; 57 kDa), have recently been reported to perform as functional homologues to *E. coli* RNase E [146]. The concerted activity of RNases J1 and J2 has been shown to cleave the *B. subtilis thrS* mRNA leader with behavior expected of an RNase E functional homolog [146]. In addition to affecting transcript specific mRNA decay, the role of J1/J2 in global mRNA turnover has recently been described [147]. Interestingly, RNase J1 also functions as a 5′ → 3′ exoribonuclease in the maturation of 16S rRNA and in regulating the mRNA stability of the *B. thuringiensis* stationary phase insecticidal protein transcript *cryIIIA* and the *trp* leader sequence [26, 27]. 

In addition to its role in tRNA processing, the *B. subtilis* ribonuclease, RNase P, has been shown to affect the mRNA stability of the adenine efflux pump transcript, *pbuE* [148]. Other ribonucleases that affect the organism's mRNA turnover are currently being sought after. Table 1 lists the ensemble of putative *B. subtilis* ribonucleases that have been identified to date. It is highly likely that as their characterization intensifies, a subset of these ribonucleases will be determined to affect mRNA stability.

As with the *B. subtilis* RNA degradation machinery, the organism's RNA binding proteins have not been fully characterized. Studies have revealed that certain RNA binding protein functions are conserved across species, whereas others are not. For instance, as in the case of *E. coli, B. subtilis* CsrA may affect mRNA degradation [149]. The protein binds to the *hag* (flagellin) transcript, inhibits translation initiation, and prevents cell motility. However, it remains to be seen whether CsrA binding effects *hag* mRNA stability [149]. Conversely, despite its importance in mediating sRNA:mRNA hybridization within *E. coli*, Hfq is not required for *B. subtilis* sRNA:mRNA duplex formation [150]. 

Likewise, the biological role(s) and mechanism of action of *B. subtilis* sRNAs have not been as extensively characterized as their *E. coli* counterparts. Nonetheless, recent work suggests that *B. subtilis* sRNAs are produced and do have regulatory functions. Silvaggi et al. found a set of sRNAs that are induced in response to sporulation [151]. Further, Heidrich et al. have shown that the *B. subtilis* sRNA, SR1, regulates expression of genes involved in arginine catabolism [150, 152]. As these and other investigators unravel the details of *B. subtilis* sRNA production, effects, and mechanism of action, it will be exciting to determine the similarities and differences among sRNAs of various bacterial species.

As described above, several studies are in progress to characterize mechanisms that alter mRNA turnover in *B. subtilis.* However, additional studies in this organism are certainly required to further characterize the components described here and identify additional factors that influence this mechanism of gene regulation. Although *B. subtilis* is considered the model gram-positive organism for studying cellular processes, it is in fact quite different from other gram-positive bacteria. For example, the organism is motile and undergoes sporulation, whereas other gram-positive bacteria, such as *S. aureus*, do not. Likewise, RNA turnover mechanisms may not be conserved across all gram-positive bacteria. As described below, mechanisms of mRNA turnover have recently been described and may play a dynamic role in virulence and adaptation to stress responses in the human pathogen *S. aureus*.

## 4. Control of *Staphylococcus aureus* Messenger RNA Degradation

As discussed above, studies from *E. coli* and *B. subtilis* have provided insight into the processes that regulate bacterial RNA stability. Until recently, this mechanism of gene regulation was largely uncharacterized within the human pathogen *S. aureus*. In this final section, we discuss emerging efforts to characterize factors that contribute to RNA turnover in this organism.

Recent studies have revealed that *S. aureus* mRNA turnover is a highly dynamic process. Indeed, during log phase growth the half-life of ~85% of *S. aureus* transcripts is ≤2.5 minutes, yet many transcripts are stabilized as cells transition to stationary phase growth; the half-life of only ~48% of mRNA species is ≤2.5 minutes (Anderson and Dunman, unpublished; [11, 22]). While the biological significance of this apparent global change in mRNA turnover is unknown, a similar phenotype has been observed following induction of heat shock, cold shock, the stringent response, acid shock, and alkaline shock responses (Anderson and Dunman, unpublished; [11]). Collectively, these results indicate that altering mRNA stability may provide a dynamic means by which *S. aureus* cells can rapidly adapt to adverse growth conditions without the need to induce de novo transcript synthesis.

**Figure 4:**
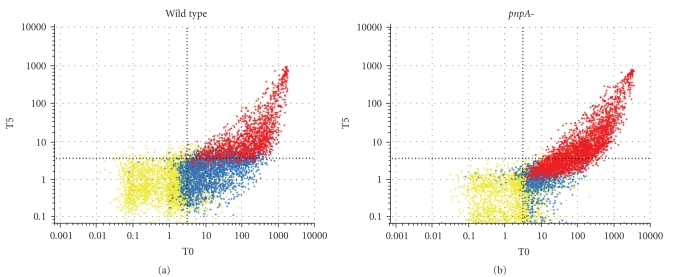
Degradation profiles of S. aureus wild type and pnpA-mutant cells. RNA signal intensity values for each GeneChip transcript are plotted at 0 minute (T0; *X*-axis) and 5 minutes (T5; *Y*-axis) posttranscriptional arrest. Red represents transcripts considered “present” in both T0 and T5 samples (Affymetrix algorithms). Yellow represents transcripts that are “absent” in both samples. Blue represents transcripts that are present in one sample but absent in the second. Grey dashed lines indicate calculated lower limit of sensitivity for each sample. Results show that following 5 minutes of transcriptional arrest, 51.1% (1287 transcripts) of mRNA species are completely degraded within wild type *S. aureus* cells. Conversely, 17.6% (444 transcripts) of mRNA species were undetectable within isogenic *pnpA*-mutant cells at 5 minutes posttranscriptional arrest, suggesting that PNPase plays a role in global *S. aureus* mRNA turnover.

Although the ribonucleases that contribute to *S. aureus* global mRNA turnover have not yet been characterized, a transcript specific ribonuclease, RNase III, has been shown to affect the mRNA decay of virulence factors [23, 24]. During the postexponential growth phase, the noncoding-like RNA molecule, RNAIII, base pairs with *spa* (protein A). This, in turn, facilitates RNase III-mediated *spa* mRNA decay [23, 24]. One additional RNase, RNase P, has been previously studied in *S. aureus* [64, 153]. As described for *E. coli,* this ribonuclease is presumably responsible for tRNA maturation but may contribute to mRNA decay as well. BLAST analyses suggest that *S. aureus* harbors at least 14 *B. subtilis* RNase homologues (Table 1; [92, 154]). At least one of these, PNPase (*pnpA*; 77 kDa), affects global mRNA turnover. As in the case for *E. coli,* disruption of the *S. aureus pnpA* gene results in a mild global change in mRNA stability (Figure 4) suggesting that other ribonucleases can overcome the loss of PNPase function. Moreover, as in *E. coli*, *S. aureus pnpA*-mutant cells demonstrate appreciable cold sensitivity when transferred to 10°C suggesting that mRNA turnover may play a significant role in the organism's ability to adapt to environmental stresses. Given the dynamic nature of *S. aureus* mRNA turnover, coupled to the importance of PNPase in cold shock adaptation, it is likely that the modulation of mRNA turnover is an important regulatory system for this organism to adapt to otherwise deleterious growth conditions.

Even less is known about *S. aureus* RNA binding proteins. Nonetheless, an Hfq homolog has been identified but in contrast to *E. coli*, *S. aureus* Hfq is not required for sRNA:mRNA duplex formation, stress adaptation, virulence, or metabolic processes [155–157]. We recently determined that the nucleic acid binding protein staphylococcal accessory regulator, SarA, may affect mRNA turnover. That study showed that a product of the *sarA* locus influences the stability of several transcripts including two surface expressed virulence factor transcripts, *spa* and *cna* (collagen adhesion protein) [22]. SarA may directly or indirectly affect mRNA stability by binding target transcripts or regulating another factor which binds mRNA, respectively.

As mentioned above, *S. aureus* produces a well-characterized noncoding-like RNA regulator, RNAIII, which modulates virulence factor transcript stability [23, 24]. The regulatory effects of RNAIII are modulated by the RNA molecule rather than the protein product of this locus [158]. Thus, there is precedence for the existence of additional *S. aureus* sRNA-like regulators. Indeed, Pichon and Felden identified twelve sRNA-like molecules that are differentially expressed among strains and in a growth phase-specific manner. Seven of these are encoded on *S. aureus* pathogenicity islands and are presumably involved in the regulation of virulence factors. Of these, one was shown, in vitro, to base pair with the 3′ UTR of an ABC transporter mRNA [156]. Although not formally evaluated, it is likely that duplex formation affects mRNA stability. Moreover, we recently found that *S. aureus* produces 139 small stable (half-lives ≥30 minutes following transcriptional arrest) RNA molecules [11, 22]. Based on their size, absence of an obvious open reading frame, and mapping of each molecule's transcriptional unit, it is likely that they constitute additional sRNA-like molecules. Nearly all of these small stable RNAs are differentially expressed in response to growth phase and stress conditions. Thus, we predict that *S. aureus* small RNAs will affect the mRNA stability of factors involved in virulence, metabolism, and adaptation to otherwise deleterious growth conditions. 

Global and transcript specific studies indicate that the regulation of gene expression by altering mRNA stability is a dynamic and previously unappreciated means of controlling gene expression in *S. aureus*. Certainly, further studies are needed to determine the function of each ribonuclease, identify the regulatory cues that mediate alterations to mRNA stability, and establish the biological significance of altering mRNA stability in this important human pathogen.

## 5. Concluding Remarks

The modulation of mRNA turnover is a recently appreciated regulatory phenomenon that spans most, if not all, bacterial species. Presumably, altering the mRNA degradation properties of individual or subsets of mRNA species allows the cell to quickly adapt to endogenous or exogenous cues without having to expend the energy required for de novo transcript synthesis. A survey of the three factors described here, ribonucleases, RNA binding proteins, and sRNAs among three genetically divergent organisms, suggests that mechanisms of modulating mRNA turnover are generally conserved across bacteria. As the field matures, it is likely that additional conserved RNA stabilization and destabilization processes will be identified. Despite these similarities, it is also obvious that species specific differences do occur. Perhaps this is most evident by the absence of a sequence homolog to the central component of the *E. coli* RNA degradosome, RNase E, among gram-positive bacteria. Clearly, further studies are required to better characterize already identified members of each organism's mRNA turnover machinery and to expand identification of previously unrecognized components. 

## Figures and Tables

**Table 1:** *E. coli, B. subtilis,* and *S. aureus* ribonucleases.

Endoribonucleases				Exoribonucleases				Toxin-mediated ribonucleases			
	Gene symbol				Gene symbol				Gene symbol		
											
	*E. coli*	*B. subtilis*	*S. aureus*		*E. coli*	*B. subtilis*	*S. aureus*		*E. coli*	*B. subtilis*	*S. aureus*
											
RNase III	*rnc*	*rncS*	*rnc*	Oligo-RNase	*orn*			RelE	*relE*		
RNase BN/Z	*elaC*	*rnz *	SA1335^†^	PNPase	*pnp*	*pnpA*	pnpA*	YoeB	*yoeB*		
RNase Bsn		*yurI*		RNase II	*rnb*			ChpBK	*chpBK*		
RNase E	*rne*			RNase D	*rnd*			MazF	*mazF *	*ydcE*	* mazF*
RNase G	*rng*			RNase PH	*rph*	*rph*					
RNase HI	*rnhA*			RNase R	*rnr*	*rnr*	rnr*				
RNase HII	*rnhB*	*rnhB*	rnhB*	RNase T	*rnt*						
RNase HIII		*rnhC*	SA0987^†^	YhaM		*yhaM*	SA1660^†^				
RNase I	*rna*										
RNase J1		*rnjA*	SA0940^†^						Putative ribonucleases		
											
RNase J2		*rnjB*	SA1118^†^						Gene symbol		
											
RNase LS	*rnlA*								*E. coli*	*B. subtilis*	*S. aureus*
											
RNase M5		*rnmV*	SA0450^†^							*yusF*	SA0768^†^
RNase P	*rnpAB*	*rnpAB*	*rnpAB*							* ymdA*	SA1129^†^

*Gene symbols of putative RNases in *S. aureus.*

^†^ *S. aureus* N315 loci of genes homologous to *B. subtilis* RNases.

**Table 2:** *E. coli* 3′ → 5′ exoribonucleases.

Ribonuclease	Gene	Size	tRNA processing mechanism	mRNA stability
PNPase	*pnp*	80 kDa	ND	yes
Oligo-RNase	*orn*	38 kDa	ND	yes
RNase D	*rnd*	42 kDa	Final processing of 3′ terminus	ND
RNase II	*rnb*	72 kDa	3′ trailer removal	yes
RNase PH	*rph*	24 kDa	Removal of +4, +3, and +2 residues after the 3′ CCA	ND
RNase R	*rnr*	95 kDa	ND	yes
RNase T	*rnt*	23 kDa	Removal of +4, +3, and +1 residues after the 3′ CCA	ND
